# The potential of corticomuscular and intermuscular coherence for research on human motor control

**DOI:** 10.3389/fnhum.2013.00855

**Published:** 2013-12-10

**Authors:** Tjeerd W. Boonstra

**Affiliations:** ^1^School of Psychiatry, University of New South WalesSydney, NSW, Australia; ^2^Black Dog InstituteSydney, NSW, Australia; ^3^MOVE Research Institute, VU UniversityAmsterdam, Netherlands

**Keywords:** coherence analysis, oscillatory activity, motor neurons, EMG, descending pathways, computational modeling

In an experimental study on the changes in descending drive during muscle fatigue, Semmler et al. (2013) investigated intermuscular (EMG–EMG) coherence in elbow muscles after eccentric exercise. They reported a broadband increase in coherence with fatigue in all elbow flexor muscle pairs and suggested that these changes reflect increased common oscillatory input to the elbow flexors. This interpretation was questioned in the Editorial by Heroux and Gandevia (2013), who noted that the mechanisms generating the reported increase in coherence are unclear and that peripheral factors such as EMG signal cancellation may provide an alternative explanation for the observed changes in coherence. Héroux and Gandevia therefore, concluded that the insights that can be obtained from investigating corticomuscular (CMC) and intermuscular (IMC) coherence are limited.

In this commentary, I will first revisited the reasons for investigating IMC and CMC. These measures have the potential to provide key insights into the composition of descending drive (Farmer, 1998) and involve non-invasive recording techniques that make them suitable for clinical applications. Subsequently, I will review current research in this area aimed to uncover the generating mechanisms of IMC and CMC. In particular, the use of computational modeling and new recording techniques are considered to obtain a more accurate estimate of the underlying changes in descending drive.

CMC and IMC can provide important insights into the diverging projects underlying motor coordination. A major open questions in motor control is how the central nervous system (CNS) controls the redundant degrees of freedom of the musculoskeletal system (Bernstein, 1967). The possibility that the CNS produces movement through the flexible combination of groups of muscles, or muscle synergies, has received much attention (Todorov, 2004; Tresch and Jarc, 2009). However, the neural implementation of such control strategies remains largely unknown. IMC is particularly suited to investigate common input to groups of muscles and can be assessed in relatively unstrained and natural task settings. Several studies have explored this notion and showed that disparate muscles, whose activity needs to be coordinated, indeed reveal significant IMC (Boonstra et al., 2009a; Poston et al., 2010; Nazarpour et al., 2012). CMC and IMC can also be used to differentiate between pathways converging onto the spinal motoneurons. Common oscillatory input underlying IMC can originate from many efferent and afferent pathways, while CMC is most likely driven exclusively by the corticospinal pathway. By comparing IMC and CMC, one may distinguish cortical from non-cortical sources of common input (Boonstra et al., 2009b).

The mechanisms underlying CMC and IMC are still being discussed and their detailed understanding would greatly enhance their research potential. Computational modeling of the involved circuitry can be used to quantify the effects and involvements of potential mechanisms. The debate about EMG rectification is a good example of the strength of such a combined approach: Using converging empirical and computation evidence the specific contribution of peripheral mechanisms such as EMG signal cancellation can be determined (Boonstra and Breakspear, 2012; Farina et al., 2013; Ward et al., 2013). Likewise, combining experimental investigations with computational modeling can be used to identify the involved neural circuitry, e.g., to specify the spinal circuitry involved in the cancellation of 10-Hz oscillations in corticospinal drive (Williams et al., 2010). Although invasive recordings are essential to validate the model design, once such a model is formulated model inversion techniques can be used to infer “hidden” model parameters (Friston et al., 2003), e.g., to infer common input from empirically observed CMC and IMC.

Computational models of CMC and IMC provide a quantitative description of the synaptic transformations of the involved pathways. CMC poses additional challenges concerning the transformation of synchronous dendritic activity (as measured by EEG) into corticospinal drive (Heitmann et al., 2013), and the innervation of corticospinal projects onto neurons at the spinal level (Harel et al., 2008). In contrast, the synaptic transformations underlying IMC are confined to the spinal motor units (MUs). They involve: (1) the transformation of presynaptic input into the firing patterns of alpha motoneurons and (2) the propagation of the action potential along the muscle fibers resulting in the MU action potential (MUAP). The MUAP itself is a composite potential and is affected by physiological changes due to exercise, fatigue, aging, and disease (McGill et al., 2001). Since changes in the MUAP and the firing rate affect the level of IMC (Boonstra and Breakspear, 2012; Farina et al., 2013), these factors need to be controlled for when comparing IMC across conditions.

New techniques may reduce some of these peripheral effects on CMC and IMC, e.g., high-pass filtering EMG before rectification may diminish the effect of EMG signal cancellation (Boonstra and Breakspear, 2012; Farina et al., 2013). In addition, high-density surface EMG (HDsEMG) has been used to improve force estimation (Staudenmann et al., 2010) and may also improve the estimation of common oscillatory input. In particular, HDsEMG signals can be decomposed into spike train patterns using advanced analysis techniques (Holobar et al., 2009). This approach inverts the second transformation concerning the MUAPs and reduces the problem to estimating the common input based on the motoneuron spike trains (Halliday et al., 1995). Assuming that the input is common to all motoneurons, this transmission is largely linear and a few motoneurons will accurately sample the input (Negro and Farina, 2011). However, physiological states like fatigue are also known to affect synaptic transmission (Gandevia, 2001), and hence affect how motoneurons sample the input. This problem may be overcome by model inversion techniques that are used to infer the common input from multiple neural spike-train data (Kulkarni and Paninski, 2007; Paninski et al., 2007). These models can be validated using experimental data where the source of common input is known, e.g., when generated by transcranial magnetic stimulation (Norton and Gorassini, 2006).

In sum, whilst the concerns expressed by Heroux and Gandevia (2013) regarding the ambiguity of the underlying mechanisms are valid, the potential of CMC and IMC to investigate new aspects in human motor control warrants further research. Novel techniques such as HDsEMG and model inversion can be used to obtain a more accurate estimate of common input and facilitate the comparison of CMC and IMC between different recording conditions.
